# Further evidence of the association between social media use, eating disorder pathology and appearance ideals and pressure: a cross-sectional study in Norwegian adolescents

**DOI:** 10.1186/s40337-024-00992-3

**Published:** 2024-02-29

**Authors:** Camilla Lindvall Dahlgren, Christine Sundgot-Borgen, Ingela Lundin Kvalem, Anne-Louise Wennersberg, Line Wisting

**Affiliations:** 1 Department of Psychology, Oslo New University College, Lovisenberggata 13, 0456 Oslo, Norway; 2 Division of Mental Health and Addiction, Regional Department of Eating Disorders, Oslo University Hospital-Ullevål, Oslo, Norway; 3 Department of Psychology, Faculty of Social Sciences, University of Oslo, Oslo, Norway

**Keywords:** Eating disorders, Appearance ideals, Appearance pressure, Social media, Instagram, TikTok

## Abstract

**Background:**

Few studies have investigated how the plethora of contemporary social media (SM) platforms relate to, and influence eating disorder (ED) pathology, appearance ideals and pressure to conform to these ideals in youth.

**Methods:**

In this study, 1558 girls (53%) and boys (47%), predominantly within the 16–19 age range, completed an online questionnaire assessing SM use and perceived influence on appearance, ED pathology, internalization of appearance ideals and perceived appearance pressure.

**Results:**

Results showed that ED pathology was common, particularly in girls, and that internalization of body ideals was gender specific, a thin ideal being more prevalent in girls, and a muscular ideal being more common in boys. Results also showed a strong association between ED pathology and perceived pressure to conform to these appearance ideals. One fourth of the participants reported spending four hours or more on SM daily, and 80% of girls reported that SM, particularly Instagram and TikTok, had a negative influence on how they felt about their appearance. These girls had significantly higher levels of ED pathology and reported higher levels of appearance pressure from the media.

**Conclusion:**

A clear pattern of associations between photo- and video specific SM platforms, ED pathology, internalization of body ideals and perceived pressure was found in this study. Adolescent girls appeared to be particularly at risk. The results illustrate an imperative need to keep addressing the potential risks of SM use in adolescents, and to continue monitoring the effect of SM on young people’s view of themselves, their appearance, and their eating habits. Future studies should attempt to identify aspects of SM use that may be particularly detrimental for girls and boys in their formative years, but also those that may enhance adolescents’ satisfaction and appreciation of their body and appearance.

*Trial registration:* The study is registered in the Open Science Framework (Identifier: 10.17605/OSF.IO/5RB6P 10.17605/OSF.IO/5RB6P).

## Abstract (plain language summary)

Social Media (SM) has become an increasingly influential part of people’s everyday life, and has transformed the patterns of communication, especially in teenagers. In this study, we investigated the relationship between eleven commonly used SM platforms in adolescents, eating disorder (ED) pathology, appearance ideals, and pressure to conform to these ideals. A little over 1500 adolescent girls and boys, predominantly within the 16-19 age range, participated in the study. Most girls who participated reported that SM use, particularly Instagram and TikTok, had a negative influence on how they felt about their appearance. These girls had significantly higher levels of ED pathology and reported higher levels of appearance pressure from the media. Future studies should attempt to identify aspects of SM use that are particularly harmful for girls and boys in their formative years, but also those who have the potential of strengthening the sense of self-worth and contribute to positive identity formation.

## Background

Social Media (SM) has become an increasingly influential part of people’s everyday life, and has transformed the patterns of communication, especially in teens [1, 2]. Recent studies show that as many as 95% of adolescents use SM [3], with Facebook, Snapchat and Instagram being some of the most popular platforms [2]. Although some studies report positive associations between SM use, psychological well-being and social support [4, 5], the majority show negative associations, especially in girls [6, 7]. Its negative influence on body image, with eating disorders (EDs) as an ultimate adverse consequence, is especially critical in teens as adolescence is a peak onset period for EDs [8].

Eating disorder pathology is characterized by negative body image, weight and shape concerns, disruptive eating patterns and low self-esteem [9]. It is common in adolescents [10] and can, if left untreated, lead to the development of clinical EDs [11]. The process through which SM negatively affects body image and ED pathology in adolescents is often described using the tripartite influence model [12]. Here, SM, in conjunct with parents and peers, constitutes one of the most important avenue for reinforcement of appearance ideals, and the pressure to comply to these standards [13]. What makes SM use especially perilous is the endless focus on appearance, the persistent feedback to one’s presentation of the physical self, and the vast opportunities for comparison, which may lead to the internalization of unattainable and unhealthy appearance ideals. Internalization of ideals is often followed by an unceasing need for self-evaluation and improvement [14, 15], sometimes leading to harmful eating behaviours and cognitions. In line with the tripartite influence model, several studies support the adverse association between SM use [16–18], the experience of appearance pressure from media [19], and the internalization of appearance ideals. The internalization of appearance ideals decrease body satisfaction [20] and increases body image concern in adolescent boys and girls [20, 21]. Also, in line with the tripartite influence model, studies report that SM use associate with [18, 22, 23] and predict [21, 24] ED pathology in adolescents.

It is the engagement with appearance-based content and activities in SM that seems to especially associate with ED pathology. The extent to which adolescents engage in these activities varies based on the SM platform they use [25, 26]. Existing evidence suggest that appearance-focused and picture sharing focused platforms (e.g. Instagram, Snapchat) compared to more text-based platforms (e.g. Twitter and Facebook) more clearly associate with elevated ED pathology [27]. Among studies on more recent platforms such as e.g. TikTok and Tinder, similar trends have been documented in young adults [28, 29]. Because the majority of studies include young adults, studies exploring if association with appearance pressure, the internalization of appearance ideals, and the severity of ED pathology differs by platform, in adolescents have been requested [30].

It is well established that adolescent boys report lower levels of body dissatisfaction and disordered eating, and higher levels of positive embodiment compared to adolescent girls [31–34]. However, findings investigating gender differences in how the use of various SM platforms are linked to appearance pressure, the internalization of body ideals, and ED symptoms, are inconclusive [18]. One study found that exposure to SM appeared to affect level and type of internalization of body ideals differently among boys and girls. In this study, the authors suggest that the distinct platform preferences among girls and boys, based on potential gender differences, might account for some of the findings [18]. Among the few studies in male samples, findings indicate that adolescent girls, more frequently than boys, use SM platforms that are visually oriented [35], which, in theory, makes them more susceptible to negative experiences on SM [17, 24, 36, 37]. Further, emerging literature suggest that adolescent boys exhibit more positive agency over their SM use, and that they use more body positive coping styles compared to girls [38]. Hypothetically, this could protect boys from negative experiences of SM use. Other studies challenge these observations by reporting that platform choices and activity participation may vary, but that disordered eating cognition and behaviour still are prevalent in both boys and girls [39, 40]. Given that few comparable studies have addressed these gender differences in adolescents, further research is essential to gain a clearer understanding and to tailor future media literacy interventions more precisely for both boys and girls [25]. Furthermore, despite a growing body of literature supporting the link between SM, ED pathology and appearance ideals and pressure, replications are essential to advance scientific understanding, particularly in exploring how these relationships manifest across different cultures and geographic regions. The primary objectives of this study were (1) to examine the utilization of various SM platforms and assess their perceived influence on appearance among adolescent girls and boys, (2) to investigate gender differences in ED pathology, appearance internalization and pressure, and time spent on SM and (3) to investigate associations and potential gender differences in time spent on SM, ED pathology, internalization of appearance ideals and pressure to conform to these ideals. The following hypothesis were formulated:

1. Girls are more likely to perceive a negative influence from SM compared to boys
2. Participants who report experiencing a negative influence from SM will exhibit significantly higher levels of ED pathology, internalization of appearance ideals and media pressure compared to participants who do not.
3. There are positive associations between ED psychopathology, internalization of the *thin/low body fat appearance ideal*, media pressure and time spent on SM in girls.
4. There are positive associations between ED psychopathology, internalization of the *muscular appearance ideal*, media pressure and time spent on SM in boys.
5. Associations between ED psychopathology, internalization of appearance ideals, media pressure and time spent on SM are significantly stronger in girls than in boys.

## Methods

### Procedure and sample

Data for this this study was gathered within the context of a broader prevalence study investigating EDs in Norwegian adolescents [34]. Over twenty upper secondary schools from five of Norway’s eleven counties were invited to participate, with a significant concentration in Oslo and Viken, both notable as the largest and among the most densely populated regions in the country. Participants in this study, predominantly within the 16–19 age range, were students from six of these schools spanning the administrative regions of Oslo, Viken, Vestfold and Telemark, and Rogaland, who accepted the invitation to take part in the research. Data collected was conducted between November 2020 and May 2021. A link to an online survey was distributed to students via their school e-mail accounts or via the schools' digital platforms. Students aged 16 years or above who provided electronic consent were eligible to participate. Students were informed that participation was voluntary, and entailed responding to questions regarding eating patterns, body and weight, loneliness, appearance attitudes and pressure, quality of life and non-suicidal self-injury (see [35, 41]). Students completed the survey either in class or at home using individual electronic devices. Prior to initiating data collection, information was made available to students, parents, and teachers via short informational videos, digital pamphlets, and communication through the schools’ digital platforms. The study was approved by the Norwegian Regional Committee for Medical and Health Research Ethics (Reference ID 116178) and the Norwegian Data Protection Authority at Oslo University Hospital. The study is registered in the Open Science Framework (Identifier: 10.17605/OSF.IO/5RB6P).

### Assessment

*Sociodemographic information.* The online survey included items on self-reported weight and height (used to calculate Body Mass Index; BMI kg/m^2^), age, gender, immigrant background, school, grade, and study programme.

*Social Media Use and Influence.* The selection of SM platforms was based on market research (iPSOS) tracking SM use in Norway in 2020. All participants were asked (Yes/No) if they were currently using one or more of the following platforms: Instagram, Snapchat, TikTok, YouTube, Pinterest, Twitter, Facebook, Reddit, Twitch, Jodel and Tinder. If participants answered “Yes,” they were also prompted to register which of the above-mentioned platform(s) they were currently using, and how much time they spent on SM daily (< 30 min, 30–60 min, 1–2 h, 2–3 h, 3–4 h, or > 4 h). Categorical responses were transformed to numbers (1–6) and scored on a Likert scale with the anchors 1 (< 30 min) and 6 (> 4 h). Participants were also asked to report (Yes/No) whether any of the SM platforms they used had a positive and/or negative influence on how they felt about their appearance («*Do any of the social media platforms you use have a positive influence on how you think about your appearance?»* and «*Do any of the social media platforms you use have a negative influence on how you think about your appearance?*»). If the response was “Yes”, participants were prompted to choose which platform(s) they felt had a positive or negative influence.

*The eating disorder examination questionnaire short (EDE-QS)* [42] is a derivative of the Eating Disorder Examination Questionnaire (EDE-Q) [43]. Comprising twelve items on a 4-point scale spanning from 0 to 3 (0 = 0 days, 1 = 1–2 days, 2 = 3–5 days, 3 = 6–7 days), the EDE-QS assesses ED cognitions and behaviors over the preceding 7 days. A sum score is calculated (Minimum = 0, Maximum = 36) where higher scores indicate higher levels of ED symptomatology. The EDE-QS has demonstrated good internal consistency, test–retest reliability, and convergent validity in individuals with probable and non-probable EDs [42, 44]. Its brevity and psychometric properties underpin the instrument’s potential as an effective screening instrument for EDs in in non-clinical samples [42]. A cut-off score of 15 is reported to represent the best trade-off between sensitivity and specificity [44]. As suggested in Prnjak et al. [44] however, a slightly lower cut-off can be applied to ensure the inclusion of participants experiencing, or likely to experience clinically significant ED symptoms. In the context of this research, a cut-off score of 13 was applied [34]. For the study’s purpose, the English version of the EDE-QS was translated to Norwegian, ensuring alignment with the Norwegian translation of the EDE-Q [45]. Cronbach’s alpha obtained in the current sample was 0.90 indicating excellent internal consistency.

*The Sociocultural Attitudes Toward Appearance Questionnaire-4 (SATAQ-4R)* [46] assesses the internalization of appearance ideals and the pressure to achieve these ideals from parents, friends, significant others, and the media. The SATAQ-4R provides separate versions for males and females, with 31 statements for females, and 28 statements for males. Responses are given on a 5-point Likert scale with the anchors Strongly Disagree and Strongly Agree. Both versions have seven subscales: (1) internalization: thin/low body fat; (2) internalization: muscular; (3) internalization: general attractiveness; (4) pressures: family; (5) pressures: media; (6) pressures: peers; and (7) pressures: significant others. A mean score is calculated for each subscale, where higher scores indicate higher internalization and perceived pressure. The Norwegian version of the SATAQ-4R has shown good psychometric properties [47]. For female adolescents, Cronbach’s alpha was 0.93, while for male adolescents it was 0.92, both indicating excellent internal consistency.

### Statistical analyses

Data was analysed using IBM SPSS Statistics 27. After testing for normality using Histograms, Normal Q-Q Plots and Kolmogorov–Smirnov statistics, between group difference were analysed using Mann–Whitney U-tests for non-parametric data, and Chi-square tests for categorical variables (Cramer’s V effect sizes were used). To test hypothesis (1) and (2), independent samples t-tests were conducted. Phi ($\mathrm{\varphi }$) (for dichotomous variables) or Pearson’s *r* (Z/$\sqrt{N}$) were used to report effect sizes, and are interpreted as 0.10 (small), 0.30 (medium) and 0.50 (large). To test hypotheses (3) and (4) correlational analyses were performed. These were reported using Spearman’s rho ($\uprho$). Spearman’s rho ($\rho$) is interpreted as 00–0.19 (very weak), 0.20–0.39 (weak), 0.40–0.59 (moderate), 0.60–0.79 (strong) and 0.80–1.0 (very strong). To test hypothesis 5), independent t-tests were performed after transforming Spearman’s rho ($\rho$) correlations into Fisher z correlations. An alpha of 0.05 was used to determine statistical significance in all analyses. In Table 5, results are reported based on data from Instagram, Snapchat, TikTok, and Pinterest, as these were the four *visual* platforms with the highest reports of negative influence among both boys (1.4–24.7%) and girls (23.9–76.4%). The other platforms were rated considerably lower in terms of negative influence, ranging from 0.5 to 5.7% in both boys and girls, and were not included in these analyses.

## Results

### Participant characteristics

A total of 1558 (53% girls, 47% boys) responded to the questionnaire. Ages ranged from 16 to 23 (*SD* = 0.95), with 99.2% (N = 1546) of the participants falling within the age range 16–19. The mean age of the total sample was 17.0 (*SD* = 0.95) years. The mean BMI score for the total sample was 21.8 (*SD* = 3.4), 21.7 (*SD* = 7.7) for girls, and 21.8 (*SD* = 3.3) for boys.

### SM platform use and perceived influence

Daily time spent on SM is reported in Table 1. A total of 99.6% of the participants reported using one or more of the listed SM platforms. Self-reported individual and cumulative SM platform use for the total sample and stratified by gender is presented in Table 2. Compared to boys, girls reported a significantly greater daily duration on SM, *X*^*2*^ (5, n = 1552) = 67.7*, p* =  < 0.001, *Cramer’s V* = 0.21. Overall, 56.7% of participants reported that one or more of the SM platforms they were currently using had a *negative* influence on how they felt about their appearance. A significantly larger proportion of these were girls (80.0%) compared to boys (29.7%), Chi-Square = 395.1, df = 1, *p* < 0.001. Perceived *negative* influence was reported as follows; Instagram (girls = 76.4%, boys = 24.7%), TikTok (girls = 55.1%, boys = 12.6%), Snapchat (girls = 23.9%, boys = 10.8%), Pinterest (girls = 29.8%, boys = 1.4%), YouTube (girls = 24.3%, boys = 8.2%), Tinder (girls = 5.7%, boys = 2.6%), Jodel (girls = 4.6%, boys = 1.0%), Facebook (girls = 3.9%, boys = 2.5%), Twitter (girls = 1.4%, boys = 1.1%), Reddit (girls = 0.5%, boys = 1.1%) and Twitch (girls = 0.5%, boys = 0.5%). Results also showed that there was a notable response overlap with regards to the number of SM platforms that elicited negative thoughts about one's own appearance. For instance, among the girls who reported perceiving a negative influence on their appearance from Instagram, 23.1% also reported experiencing a negative influence from Snapchat. Furthermore, the proportion of girls reporting negative appearance influences from Instagram, Snapchat, and TikTok collectively was 18.7%, which decreased to 8.7% when Pinterest was included in the analysis. The corresponding rates for boys were 8.5%, 4.9% and 0.7%. A total of 55.8% reported that SM had a positive influence on how they felt about their appearance. A significantly larger proportion of these were girls (61.7%) compared to boys (49.7%), Chi-Square = 22.6, df = 1, *p* < 0.001. Perceived positive influence was reported as follows; TikTok (girls = 39.6%, boys = 22.3%), Instagram (girls = 32.2%, boys = 29.9%), Snapchat (girls = 17.6%, boys = 24.1%), YouTube (girls = 16.4%, boys = 22.3%), Pinterest (girls = 8.5%, boys = 1.0% Tinder (girls = 3.7%, boys = 2.3%), Jodel (girls = 1.0%, boys = 2.1%), Facebook (girls = 1.6%, boys = 4.4%), Twitter (girls = 1.2%, boys = 1.6%), Reddit (girls = 0.4%, boys = 4.5%) and Twitch (girls = 0.8%, boys = 3.0%). Among the girls who reported perceiving a *positive* influence on their appearance from Instagram, 12.7% also reported experiencing a positive influence from Snapchat. Furthermore, the proportion of girls reporting positive appearance influences from Instagram, Snapchat, and TikTok collectively was 7.1%, which decreased to 1.9% when Pinterest was included in the analysis. The corresponding rates for boys were 17.0%, 9.6% and 0.5%.

**Table 1:** Self-reported daily time spent on SM (N = 1552)

	< 30 min (%)	30–60 min (%)	1–2 h (%)	2–3 h (%)	3–4 h (%)	> 4 h (%)
Total sample (%)	0.8	5.2	15.3	28.5	25.1	25.3
Girls (%) (n = 827)	0.4	3.4	10.2	28.7	26.7	30.7
Boys (%) (n = 725)	1.2	7.2	21.1	28.3	23.2	19.0

*SM* social media

**Table 2:** Percentage of self-reported individual and cumulative SM platform use for the total sample, and stratified by gender

	Visual SM platforms					Textual/other SM platforms					
	Snapchat	Instagram	TikTok	Pinterest	Tinder	YouTube	Facebook	Jodel	Twitter	Reddit	Twitch
Total sample	93.8%	90.8%	75.0%	57.2%	10.6%	85.9%	55.6%	26.6%	19.0%	14.1%	13.7%
Cumulative frequency	–	88.5%	70.7%	28.9%	3.4%	–	48.8%	17.3%	4.9%	1.80%	2.3%
Girls (n = 828)	97.9%	96.1%	84.7%	7.5%	13.4%	77.8%	60.4%	33.2%	12.4%	3.5%	3.7%
Cumulative frequency	–	94.7%	81.6%	49.1%	6.0%	–	49.0%	21.0%	3.4%	0.4%	0%
Boys (n = 730)	89.2%	84.7%	64.1%	34.0%	7.4%	95.1%	50.3%	19.0%	26.4%	26.3%	25.1%
Cumulative frequency	–	81.5%	58.4%	6.0%	0.7%	–	48.5%	13.2%	6.6%	3.4%	2.3%

SM use is reported in descending order, with the highest frequency reported first, followed by the next highest frequency, and so forth. The cumulative frequency is reported sequentially; for instance, frequencies reported in the Instagram column include those for Snapchat and Instagram, while frequencies in the TikTok column encompass combined frequencies for Snapchat, Instagram, and TikTok, and so forth

*SM* social media

### ED pathology

The mean EDE-QS score in the total sample was 6.7 (*SD* = 7.0), 9.5 (*SD* = 7.7) in girls, and 3.6 (*SD* = 4.3) in boys. In total, 19.9% (33.2% girls, 4.8% boys) scored at or above the EDE-QS clinical cut-off. Girls accounted for a significantly greater proportion of clinical EDE-QS scores compared to boys, X^2^(1, n = 1557) = 194.5, *p* =  < 0.001, φ = 0.36. EDE-QS scores were also significantly higher in girls (*Mdn* = 7.0) compared to boys (*Mdn* = 2.0), U(N_females_ = 828, N_males_ = 729) = 148,372.5, *Z* = − 17.4, *p* =  < 0.001.

### Associations between ED pathology, internalization of appearance ideals and pressure, and time spent on SM

Assessment outcomes and gender comparisons for age, EDE-QS and SATAQ-4R scores are reported in Table 3**.** Girls scored significantly higher than boys on most SATAQ-4R subscales. The exception was the Internalization: Muscular subscale where boys scored significantly higher than girls. As for perceived pressure, girls scored significantly higher than boys on the two subscales family and media. Associations between ED pathology, internalization of appearance ideals and appearance pressure is reported in Table 4. Results showed significant positive correlations between ED pathology and internalization of appearance ideals with effect sizes ranging from 0.24 (weak; Muscular) to 0.73 (strong; Thin/Low Body Fat) in girls. In boys, corresponding effect sizes were weak, ranging from 0.34 (Muscular) to 0.39 (Thin/Low Body Fat). The association between appearance pressure and ED pathology ranged from 0.44 (moderate; Family to 0.61 (strong; Media) in girls, and from 0.31 (weak; Family) to 0.42 (moderate; Media) in boys. Time spent on SM was significantly, albeit very weakly correlated with ED pathology in both girls (Spearman’s rho = 0.12) and boys (Spearman’s rho = 0.15). There were no significant gender differences in the association between time spent on SM and EDE-QS scores, or time spent on SM and media pressure. However, the association between time spent on SM and internalization of the Thin/Low Body Fat ideal was significantly stronger in girls than in boys (*p* < 0.01). The opposite was true for the association between time spent on SM and internalization of the Muscular ideal, which was significantly stronger in boys than compared to girls (*p* < 0.05). Also, the association between EDE-Q scores and media pressure was significantly higher in girls than in boys (*p* < 0.01).

**Table 3:** Assessment outcomes and gender comparisons: Age, ED psychopathology (EDE-QS) and internalization of appearance ideals and pressure (SATAQ-4R) (N = 1558)

	Total		Girls			Boys			z	*p*	*r*
	N	Mean *(SD)*	N	Mean *(SD*)	*Md*	N	Mean *(SD*)	*Md*			
Age	1558	17.0 (.9)	828	17.0 (.9)	17.0	730	17.1 (1.0)	17.0	− 0.68	0.495	–
EDE-QS	1557	6.7 (7.0)	828	9.5 (7.7)	7.0	729	3.6 (4.3)	2.0	− 17.40	< 0.001	0.44
SATAQ-4R											
Internalization: thin/low body fat	1556	2.6 (1.3)	827	3.3 (1.1)	3.3	729	1.8 (.9)	1.5	− 23.48	< 0.001	0.60
Internalization: muscular	1556	2.9 (1.0)	827	2.5 (.7)	2.4	729	3.5 (1.0)	3.5	− 20.74	< 0.001	0.53
Internalization: general attractiveness	1555	3.8 (1.0)	828	4.3 (.6)	4.3	727	3.3 (1.1)	3.5	− 17.78	< 0.001	0.45
Pressures: family	1556	2.0 (0.9)	827	2.2 (1.0)	2.0	729	1.9 (.7)	1.6	− 4.79	< 0.001	0.12
Pressures: peers	1552	2.4 (1.1)	824	2.4 (1.1)	2.3	728	2.4 (1.1)	2.5	− 0.74	0.457	–
Pressures: significant others	1552	1.8 (1.0)	826	1.9 (1.0)	1.5	726	1.7 (.9)	1.2	− 0.82	0.069	–
Pressures: media	1556	2.8 (1.3)	827	3.4 (1.2)	3.8	729	2.1 (1.1)	1.8	− 19.91	< 0.001	0.50

Effect size (i.e., *r)* coefficients are only reported where there is a significant group difference

*ED* eating disorder, *EDE-QS* eating disorder examination questionnaire short, *SATAQ-4R* Sociocultural Attitudes Towards Appearance-4-Revised, *Md* Median

**Table 4:** Spearman’s rho (${\varvec{\rho}}$) correlations among EDE-QS scores, SATAQ-4R scores and daily time spent on SM in girls and boys

	BMI (kg/m^2^)	EDE-QS (Mean)	Time spent on SM	SATAQ-4R (I) thin/low body fat	SATAQ-4R (I) muscular	SATAQ-4R (I) general attr	SATAQ-4R (P) family	SATAQ-4R (P) peers	SATAQ-4R (P) sign. others	SATAQ-4R (P) media
Girls (n = 792)										
BMI (kg/m^2^)	–	0.30**	0.56	0.18**	0.16	0.07	0.28**	0.22**	0.25**	0.21**
EDE-QS (Mean)	0.30**	–	0.12**	0.73**	0.24**	0.50**	0.44**	0.60**	0.51**	0.61**
Time spent on SM	0.56	0.12**	–	0.17**	0.00	0.21**	0.21**	0.16**	0.11**	0.11*
Boys (n = 706)										
BMI (kg/m^2^)	–	0.22**		0.15**	0.20**	0.03	0.07	0.08*	0.10**	0.08*
EDE-QS (Mean)	0.22**	–	0.15*	0.39**	0.34*	0.35**	0.31**	0.36**	0.40**	0.42**
Time spent on SM		0.15*	–	0.03	0.10**	0.16**	0.06	0.14**	0.12**	0.13**

Spearman’s rho correlations are reported. Correlation is significant at the 0.05* and 0.01** level (2-tailed)

*BMI* body mass index, *EDE-QS* eating disorder examination questionnaire short (the total mean score is used), *SM* social media, *SATAQ-4R* Sociocultural Attitudes Towards Appearance-4-Revised, (*I)* internalization subscale, *(P)* pressure subscale.

### Associations between perceived positive and negative influence of SM use, internalization of appearance ideals, pressure, and ED pathology

Participants who reported “Yes” to the question “Do any of the specific SM platforms you use have a *negative* influence on how you feel about your appearance” scored significantly higher (M = 9.1, *SD* = 7.5) on the EDE-QS compared to participants who did not responded “Yes” (M = 3.7, *SD* = 4.8), *t*(1466) = 18.5, *p* =  < 0.001. The effect size was large (*d* = 0.9). There were no significant differences in EDE-QS scores between those who did, or did not responded “Yes” to the question “Do any of the specific SM platforms you use have a *positive* influence on how you feel about your appearance”.

In Table 5, EDE-QS scores, SATAQ-4R scores and time spent on SM are compared based on whether the participants did, or did not respond “Yes” to the question “*Do any of the social media platforms you use have a negative influence on how you feel about your appearance*”. A “No” response in Table 5 indicates the *lack of affirmation* that the SM platform has a negative influence on the participants perception of her/his appearance. The four visual platforms that were ranked highest in terms of perceived negative influence were chosen for these analyses. Overall, participants who responded “Yes” had significantly higher EDE-QS and SATAQ-4R scores compared to those who did not responded “Yes”. An inconclusive pattern regarding the relationship between time spent on SM and reports of negative influence emerged across the four visual SM platforms.

**Table 5:** Mann–Whitney U test comparing Yes and No* responses to whether Instagram, Snapchat, TikTok or Pinterest has a negative influence on ED pathology, internalization of appearance ideals, media pressure and daily social media use in girls and boys

Means (SD)	Instagram				Snapchat				TikTok				Pinterest			
	Yes (n = 633)	No* (n = 195)	*p*	Z	Yes (n = 198)	No*N = 630)	*p*	Z	Yes (n = 456)	No* (n = 372)	*p*	Z	Yes (n = 247)	No* (n = 581)	*p*	Z
Girls																
EDE-QS^1^	10.5 (7.7)	6.5 (6.8)	< 0.001	− 7.2	12.5 (8.3)	8.6 (7.3)	< .001	− 5.8	11.2 (7.9)	7.5 (6.9)	< 0.001	− 7.3	11.0 (7.7)	8.9 (7.6)	< 0.001	− 4.0
SATAQ-4R Thin/Low Body Fat^2^	3.4 (1.0)	2.9 (1.1)	0.001	− 6.1	3.6 (1.1)	3.2 (1.1)	< 0.001	− 4.9	3.5 (1.0)	3.0 (1.1)	< .001	− 6.1	3.6 (1.0)	3.2 (1.1)	< 0.001	− 4.2
SATAQ-4R Muscular^2^	2.5 (.7)	2.3 (.8)	< 0.001	− 3.2	2.6 (0.7)	2.4 (0.7)	.007	− 2.7	2.5 (0.7)	2.4 (0.7)	0.002	− 3.1	2.5 (0.7)	2.4 (0.7)	0.268	− 1.1
SATAQ-4R General attr.^2^	4.3 (0.6)	3.9 (0.7)	< 0.001	− 7.6	4.4 (0.6)	4.2 (0.6)	< 0.001	− 4.3	4.4 (0.6)	4.1 (0.7)	< 0.001	− 7.6	4.4 (0.5)	4.1 (0.6)	< 0.001	− 5.5
SATAQ-4R Pressures: Media^2^	3.7 (1.0)	2.5 (1.1)	< 0.001	− 12.6	4.0 (1.0)	3.2 (1.1)	< 0.001	− 7.8	3.8 (1.0)	3.0 (1.1)	< 0.001	− 10.8	3.8(1.0)	3.2 (1.2)	< 0.001	− 7.0
SM use (time)	5.8 (1.0)	5.5 (1.3)	.021	− 2.3	5.8 (1.1)	5.7 (1.1)	.449	− 7.6	5.9 (1.0)	5.5 (1.2)	< 0.001	− 4.3	5.8 (1.1)	5.7 (1.1)	.018	− 2.4

Means (SD)	Instagram				Snapchat				TikTok				Pinterest			
	Yes (n = 179)	No* (n = 550)	*P*	Z	Yes (n = 79)	No* (n = 650)	*p*	Z	Yes (n = 91)	No* (n = 638)	*p*	Z	Yes (n = 10)	No* (n = 729)	*p*	Z
Boys																
EDE-QS^1^	5.4 (5.2)	3.0 (3.8)	< .001	− 7.1	6.4 (5.7)	3.3 (4.0)	< .001	− 5.9	5.7 (4.9)	3.3 (4.2)	< .001	− 6.0	6.9 (6.5)	3.6 (4.3)	0.025	− 2.2
SATAQ-4R Thin/Low Body Fat^2^	2.0 (1.0)	1.8 (0.9)	0.001	− 3.2	2.1 (1.1)	1.8 (0.9)	0.016	− 2.4	2.2 (1.1)	1.8 (0.9)	< .001	− 3.5	2.8 (1.2)	1.8 (0.9)	0.102	− 2.5
SATAQ-4R Muscular^2^	3.7 (0.8)	3.4 (1.0)	0.001	− 3.3	3.8 (0.9)	3.5 (1.0)	0.005	− 2.8	3.7 (0.7)	3.5 (1.0)	0.022	− 2.3	3.8 (0.6)	3.5 (1.0)	.374	− 0.9
SATAQ-4R General attr.^2^	3.7 (0.9)	3.2 (1.1)	< .001	− 5.8	3.9 (0.8)	3.3 (1.1)	< .001	− 4.8	3.9 (0.8)	3.3 (1.1)	< .001	− 5.2	3.8 (0.9)	3.3 (1.1)	.171	− 1.4
SATAQ-4R Pressures: Media^2^	3.0 (1.0)	1.8 (1.0)	< .001	− 12.7	3.2 (1.0)	2.0 (1.0)	< .001	− 9.1	3.3 (1.0)	2.0 (1.0)	< .001	− 10.1	3.6 (0.7)	2.1 (1.1)	< .001	− 4.0
SM use (time)	5.4 (1.1)	5.2 (1.3)	0.128	− 1.5	5.6 (1.1)	5.2 (1.3)	0.005	− 2.8	5.5 (1.1)	5.2 (1.3)	0.008	− 2.6	5.6 (1.8)	5.2 (1.2)	0.239	− 1.2

*ED* eating disorder, *BMI* body mass index, *EDE-QS* eating disorder examination questionnaire short, *SATAQ-4R* Sociocultural Attitudes Towards Appearance-4-Revised. ^1^ = EDE-QS mean scores and standard deviation (SD) are reported, ^2= ^SATAQ-4R subscale mean scores and standard deviations are reported. Between group difference were analysed using Mann–Whitney U-tests for non-parametric data. ^*^ = A “No” response indicates a lack of affirmation that the SM platform has a negative influence on the participants perception of her/his appearance

## Discussion

In line with existing literature on gender differences in ED pathology [48], our results showed significantly higher levels of ED pathology in girls compared to boys. Our results also concur with previous studies [49, 50] reporting higher levels of internalization of appearance ideals in girls compared to boys, the one exception being the muscular ideal where boys scored significantly higher. Our results also align with studies seen in American adolescents [46], supporting that internalization of the western ideal and perceived appearance pressure represents a phenomenon valid across geographic regions and cultures.

In the current study, we found significant, strong positive associations between ED pathology, internalization of body ideals, and the perceived pressure from family, peers, significant others, and media in both girls and boys. These patterns of associations align with research in young adult samples, including college females [46], Turkish college females [51], and Italian males and females (18–72 years) [49]. However, associations between internalization of appearance ideals, especially the thin ideal and ED pathology were consistently stronger in girls. As for the association between ED pathology and appearance pressure, the strongest associations were found on the media subscale, both in girls and boys. Notably, the link between ED pathology and media pressure was significantly stronger in girls, indicating that girls may be more vulnerable to the adverse effects of SM on eating cognitions and behaviors. Conversely, it is plausible that girls with problematic eating behaviors are more inclined to perceive pressure from media to adhere to thin beauty standards.

Nearly all adolescents in the current study reported using SM. However, girls and boys had different patterns with regards to what SM platforms they used, and how much time they spentd on these daily. Snapchat, Instagram, TikTok, and YouTube were the most frequently used platforms, supporting previous research showing that picture and video sharing platforms are most popular in adolescents to date [2]. Moreover, nearly two thirds of the girls reported spending three or more hours on SM daily. The corresponding time spent for boys was markedly lower. Notably, a substantial proportion of girls reported that SM adversely affected their self-perception of appearance, particularly on photo and video sharing platforms like Instagram and TikTok. These findings resonate with a systematic review [52] that highlights a strong correlation between SM usage and ED pathology. The heightened exposure to appearance ideals, coupled with potential unfavourable comparisons of one’s own appearance, may contribute to body dissatisfaction and subsequently, elevated ED pathology levels. The core feature of both Instagram and TikTok is to share and view picture and video-based content, which is often edited and filtered to make it more attractive [53]. These may generate adverse social comparisons leading to body dissatisfaction and low self-esteem, well-known risk factors for the development of EDs. A recent literature review [54] suggests that highly visual SM platforms are of particular relevance for ED risk among adolescents as these may enhance upward comparison and normalize harmful eating behaviors and cognitions. In fact (pre)adolescents have been found to be particularly vulnerable for adverse outcomes of SM usage, with research showing associations between time spent on SM and the risk of developing mental health problems [55]. In a recent meta-analysis, [56] excessive time spent on various SM platforms correlated with an increase in anxiety and depression symptoms during the Covid-19 pandemic, the period during which the current study was conducted. In our study, a notable albeit weak association was observed between time spent on SM and ED symptoms in both male and female adolescents. However, we did not specifically investigate the details of SM use, e.g., the extent to which the adolescents viewed, shared, liked and/or in other ways engaged in content relevant to appearance ideals, pressure, and ED pathology. Given that studies have shown that certain activities including viewing, manipulating, uploading photos, appearance comparisons, and seeking feedback [14, 23], may play a pivotal role in the observed association between SM use and ED pathology in adolescents [57, 58], this represents a crucial area for future research explorations.

Both Instagram and TikTok algorithms tailor content to each user based on previous online activity. Such algorithms may function like a vicious cycle as users, once they have started using harmful content, will experience a surge of similar content. Once a SM feed becomes saturated with content focused on body image, weight, and food content, disentangling from such content becomes highly challenging. According to Thomas [59] replacing content in ones SM feed requires an active and conscious effort over time, and may help explain why many adolescents persistently engage with content that adversely affect their self-perception, self-esteem and sense of self-worth. This apparent paradox emphasizes the profound influence SM can exert on impressionable adolescents and illuminates a critical dilemma; the very features tailored to attract and retain SM users to bolster profitability and success of the entities behind these platforms, concurrently undermine the well-being of their users. The present study underscores the significance of this detrimental cycle, particularly among girls. A striking 80% of adolescent girls in our sample reported that SM adversely affects their self-perception of appearance. Concurrently, nearly a third of these girls reported dedicating four hours or more to SM daily. When considering the unprecedented rise in SM use during the Covid-19 pandemic [60] and its potential link to the increasing incidence of EDs in adolescent girls [34, 61, 62, 63], this trend becomes deeply concerning.

When discussing the negative influence of visual SM platforms on ED psychopathology and appearance, it is worth noting that a substantial proportion of both girls (61.7%) and boys (49.7%) reported that SM had a positive influence on how they felt about their appearance. Content celebrating diverse appearances [64], body-positive profiles and communities [65], “SM vs. reality” images have shown to be associated with improvements in young women’s positive mood, body satisfaction and body appreciation, and have the potential to bolster women’s body satisfaction [66]. However, further research is required to evaluate potential long-term positive outcomes and to determine how best to incorporate them into targeted prevention initiatives. While considering prevention and intervention strategies for EDs in adolescents, enhancing SM literacy and addressing harmful beauty standards via cognitive dissonance methods in selective interventions appears as a paramount first step [67, 68]. The Body Project [69] a preventive initiative designed to counteract the pervasive pursuit of the thin beauty ideal, stands as one of the most rigorously documented prevention programs globally. Currently, its efficacy is under evaluation in a randomised controlled trial in Norway [70] emphasizing the program’s potential applicability and relevance across diverse cultural and regional settings in adolescent cohorts.

Although causal inferences cannot be definitively drawn, it is likely that a bidirectional relationship exists between SM use and ED pathology. Specifically, among girls reporting negative influences from picture and video-based platforms, significantly elevated levels of ED pathology, as well as heightened internalization and pressure regarding appearance, were observed. Moreover, participants who reported that the SM platforms they were using had a negative influence on how they felt about their appearance also reported spending more time on SM daily. Further, positive associations between time spent on SM, ED pathology and appearance ideals and pressures were observed among both girls and boys. The exception for girls was the muscular ideal, and for boys, the exception was internalization of the thin/low body fat ideal. The association between SM use, appearance, and ED pathology has been illustrated in several other studies [36, 40]. A recent review [54] reported that the strongest link between SM use and ED risk in adolescents can be found among those who are high investors in photo-based platforms. Further, highly visual SM platforms such as Instagram and TikTok have shown to be strongly associated with ED risk factors such as body image concerns and body dissatisfaction [71]. Although most existing studies are cross-sectional precluding causal inferences, a longitudinal study in adolescents [21] showed that SM use does in fact lead to body dissatisfaction, rather than those with body image concerns seeking increased SM use.

### Strengths and limitations

The strengths of this study are a large adolescent sample consisting of both girls and boys, the use of validated assessment tools, and the inclusion of a wide range of SM platforms commonly used by adolescents. However, there are some limitations and methodological aspects to consider when interpreting the results. Given that data was collected during the Covid-19 pandemic, one should account for the increase in SM use during this period, which is likely to have influenced the internalization of, and perceived pressure to align with prevailing beauty standards. This, in turn, may have had an adverse effect on the onset and/or intensification of ED psychopathology in adolescents. In addition, the response options regarding the influence of SM on appearance was dichotomous (Yes/No), and the cross-sectional design hinders inferences about cause and effect. It's essential to note that our BMI calculations did not adjust for age. Additionally, the SATAQ-4R focuses on general media pressure without specifically addressing social media pressure. While the EDE-QS is developed and validated in samples that to an extent overlaps with our study's sample, it should be noted that the Gideon et al. [42] and Prjnak et al. [44] studies involve older participants and a higher proportion of females compared to our current study. Whereas prior studies [72, 73] have suggested that a lower threshold may be required to identify clinically significant ED symptoms in males than in females, more research is needed to identify an appropriate EDE-QS cut-off in both adult and adolescent populations in the community. In addition, altering the ED pathology threshold can have produced different proportions compared to previous studies.

### Implications

Our results illustrate the critical importance to keep addressing the potential risks associated with SM use among adolescents, and to maintain vigilance in monitoring how SM influence young people’s self-perception, body image, and eating behaviors. Additionally, our study highlights the need to incorporate content related to SM in ED prevention programs. Such programs should focus on promoting awareness of how an excessive emphasis on appearance on SM can contribute to body dissatisfaction and negative self-comparisons among adolescents and their peers. Such awareness can play a pivotal role in reducing the risk of developing unhealthy relationships with food, weight, and body image, thereby contributing to the prevention of eating disorders in this high-risk group. Moreover, in line with previously published recommendations [68], our results emphasize the necessity of enhancing media literacy and increasing awareness about both the positive and negative aspects of SM usage. Social media, when harnessed thoughtfully, can serve as a powerful tool in increasing ED literacy, promoting healthy eating behaviors and fostering positive body image. An inspiring example of leveraging SM for positive change is the pioneering Norwegian TikTok series “TOXIC” [74]. It effectively utilizes a platform widely used by adolescents to increase awareness about EDs in boys and encourages individuals to speak up and seek help. This initiative exemplifies the potential to counteract the negative influence of SM when used thoughtfully and constructively and could serve as a source of inspiration for researchers, clinicians and ED advocacy groups alike.

## Data Availability

The data that support the findings of this study are available on reasonable request from the corresponding author, [CLD]. The data are not publicly available due to containing information that could compromise the privacy of research participants.

## Abbreviations

BMI: Body mass index
ED: Eating disorder
EDE-QS: Eating Disorder Examination Questionnaire Short Version
SATAQ-4R: Sociocultural Attitudes Towards Appearance Questionnaire 4 Revised
SM: Social media
